# Attention-deficit hyperactivity disorder shares copy number variant risk with schizophrenia and autism spectrum disorder

**DOI:** 10.1038/s41398-019-0599-y

**Published:** 2019-10-17

**Authors:** Olafur O. Gudmundsson, G. Bragi Walters, Andres Ingason, Stefan Johansson, Tetyana Zayats, Lavinia Athanasiu, Ida Elken Sonderby, Omar Gustafsson, Muhammad S. Nawaz, Gudbjorn F. Jonsson, Lina Jonsson, Per-Morten Knappskog, Ester Ingvarsdottir, Katrin Davidsdottir, Srdjan Djurovic, Gun Peggy Strømstad Knudsen, Ragna Bugge Askeland, Gyda S. Haraldsdottir, Gisli Baldursson, Pall Magnusson, Engilbert Sigurdsson, Daniel F. Gudbjartsson, Hreinn Stefansson, Ole A. Andreassen, Jan Haavik, Ted Reichborn-Kjennerud, Kari Stefansson

**Affiliations:** 1 deCODE genetics/Amgen, Reykjavík, Iceland; 2 Faculty of Medicine, University of Iceland, Reykjavík, Iceland; 3 Department of Child and Adolescent Psychiatry, National University Hospital, Reykjavik, Iceland; 4 Department of Clinical Science, University of Bergen, Bergen, Norway; 5 Department of Medical Genetics, Haukeland University Hospital, Bergen, Norway; 6 K.G. Jebsen Centre for Neuropsychiatric Disorders, Department of Biomedicine, University of Bergen, Bergen, Norway; 7 NORMENT, K.G. Jebsen Centre for Psychosis Research, Institute of Clinical Medicine, University of Oslo and Division of Mental Health and Addiction, Oslo University Hospital, Oslo, Norway; 8 Department of Pharmacology, Institute of Neuroscience and Physiology, Sahlgrenska Academy, University of Gothenburg, Gothenburg, Sweden; 9 The Centre for Child Development and Behaviour, Capital Area Primary Health Care, Reykjavik, Iceland; 10 Department of Medical Genetics, Oslo University Hospital, Kirkeveien 166, 424, Oslo, Norway; 11 Department of Mental Disorders, Norwegian Institute of Public Health, P. O. Box 4404 Nydalen, 0403 Oslo, Norway; 12 Department of Psychiatry, National University Hospital, Reykjavík, Iceland; 13 School of Engineering and Natural Sciences, University of Iceland, Reykjavik, Iceland; 14 Division of Psychiatry, Haukeland University Hospital, Bergen, Norway; 15 Institute of Clinical Medicine, University of Oslo, Oslo, Norway

**Keywords:** Comparative genomics, ADHD

## Abstract

Attention-deficit/hyperactivity disorder (ADHD) is a highly heritable common childhood-onset neurodevelopmental disorder. Some rare copy number variations (CNVs) affect multiple neurodevelopmental disorders such as intellectual disability, autism spectrum disorders (ASD), schizophrenia and ADHD. The aim of this study is to determine to what extent ADHD shares high risk CNV alleles with schizophrenia and ASD. We compiled 19 neuropsychiatric CNVs and test 14, with sufficient power, for association with ADHD in Icelandic and Norwegian samples. Eight associate with ADHD; deletions at 2p16.3 (*NRXN1*), 15q11.2, 15q13.3 (BP4 & BP4.5–BP5) and 22q11.21, and duplications at 1q21.1 distal, 16p11.2 proximal, 16p13.11 and 22q11.21. Six of the CNVs have not been associated with ADHD before. As a group, the 19 CNVs associate with ADHD (OR = 2.43, *P* = 1.6 × 10^−21^), even when comorbid ASD and schizophrenia are excluded from the sample. These results highlight the pleiotropic effect of the neuropsychiatric CNVs and add evidence for ADHD, ASD and schizophrenia being related neurodevelopmental disorders rather than distinct entities.

## Introduction

Attention-deficit/hyperactivity disorder (ADHD) is a common childhood-onset neurodevelopmental disorder characterized by a triad of signs—age-inappropriate levels of inattentive, hyperactive and impulsive behavior—that lead to severe impairments^1^. ADHD is estimated to affect 3.4% of the population worldwide^2^. Follow-up studies of children have documented the persistence of ADHD symptoms into adulthood in approximately two-thirds of patients^3^.

Studies have focused on the relationship between ADHD and schizophrenia^4^ in light of considerable overlap in clinical presentation and because ADHD is diagnosed in a high proportion of children at genetic risk of schizophrenia^5^. Also, a schizophrenia polygenic score, estimated from an adult population, was found to confer a small but significant risk of childhood ADHD^6^. A history of ADHD signs is common in individuals who develop schizophrenia, with attentional impairment as a central cognitive feature of both disorders^4^. Deficits in working memory, cognitive flexibility and attention seen in ADHD are similar to those observed in schizophrenia^7^. Likewise family-based and twin studies in clinical ADHD samples have shown that signs of autism spectrum disorders (ASD) are common within ADHD families and that more than half of the phenotypic variance in either disorder may be attributable to shared genetic factors^8^. This is further supported by analyses showing that some sequence variants confer risk of both ADHD and ASD^9,10^ and the two disorders share considerable common variant genetic overlap^11^.

Heritability estimates suggest that ~74% of the phenotypic variability in ADHD is due to variants in the sequence of the germline genome^12^. Genome-wide association studies (GWAS) have only recently reached sample sizes with adequate power to yield variants significantly associated with ADHD^13^. The genome-wide single-nucleotide polymorphism (SNP) heritability of ADHD has been estimated at 22%, corresponding to the contribution of common SNPs to ADHD susceptibility^13^. This is lower than the heritability estimated from twin studies and suggests that rare variants may also contribute to the risk of ADHD^14^.

Rare copy number variations (CNVs) have been associated with cognitive deficits that can result in disadvantages in educational attainment and various adult life outcomes but more directly with increased risk of psychiatric and developmental disorders, including schizophrenia, ADHD, ASD and developmental delay^15–20^. Evidence for increased burden of large, rare CNVs in ADHD have been reported, suggesting CNVs contribute to the disorder^21–25^. Two CNVs have previously been associated with ADHD, 16p13.11 duplications^23^ and 22q11.21 deletions^23,26^. Furthermore, ADHD has been reported in individuals with 1q21.1 distal deletions^23^ or duplications^27^, 15q11.2 deletions^28^, 15q13.3 deletions^29,30^ and 16p11.2 proximal deletions^31^ or duplications^32^, but have not been statistically tested for association.

While much of the current knowledge on the effects of CNVs and associated risk comes from schizophrenia and ASD samples, the aim of this study was to determine to what extent rare CNVs, previously associated with schizophrenia and/or ASD, associate with ADHD in a combined Icelandic and Norwegian population sample.

## Materials and methods

### Samples

#### Icelandic sample

This study was approved by the National Bioethics Committee of Iceland. All individuals signed an informed consent prior to giving a blood or buccal sample. Social security numbers of participants were encrypted through a process overseen by the Data Protection Authority before being analyzed^33^.

The ADHD affected (total *N* = 5650) were included on the basis of meeting criteria for ADHD diagnosis, any Diagnostic and Statistical Manual of Mental Disorders (DSM-IV) and International Classification of Diseases (ICD-9 or −10)-based subtype (*N* = 2665, male *N* = 1746) or on confirmed information on treatment with ADHD medication according to the Directorate of Health centralized medication database (*N* = 2985, male *N* = 1489) (Supplementary Table 1).

Participants were diagnosed by a psychiatrist or pediatrician, in settings of collaborating centers, the University hospital, the Centre for Child Development and Behavior, the SLF’s Rehabilitation Center and private practice clinics with diagnoses made on the basis of standardized diagnostic assessments, reviewed by experienced clinicians, as part of their diagnostic and treatment regimen in the national healthcare service, and not specifically for this study.

The participants identified in the Directorate of Health medication database have all been prescribed medication from the centrally acting sympathomimetic (N06BA) class of drugs (amphetamine (01), methylphenidate (04) or atomoxetine (09)), based on the Anatomical Therapeutic Chemical (ATC) Classification System. However, as diagnoses are not registered in the medication database, information on the ADHD status was not available for these individuals. Although, amphetamine can be prescribed for signs and symptoms other than ADHD, individuals prescribed amphetamine make up only 1% of the entire ADHD medication sample and none of them are neuropsychiatric CNV carriers. As methylphenydate and atomoxetine are exclusively prescribed for ADHD in Iceland, we have assumed that the individuals that make up this group have all sought treatment for the signs and symptoms of ADHD.

While diagnosed male subjects are about twice as many as diagnosed female subjects, the gender ratio is close to 1:1 within the group prescribed medication, and so overall the male-to-female ratio among ADHD subjects is about 3:2. The control sample (*N* = 155,122, male *N* = 71,492) was recruited through various projects at deCODE genetics.

#### Norwegian samples

The Norwegian ADHD affected (*N* = 3233) were obtained from the Norwegian Mother and Child cohort study (Mor og Barn; MoBa)^34,35^, which includes children (*N* = 1858 born between 1999 and 2009) and adults (*N* = 941; ADHD affected adults who are not parents of ADHD affected children), and from the Bergen adult ADHD study (*N* = 434)^36^. Norwegian controls were children (*N* = 8245) and adults (*N* = 19,316) from the MoBa study, controls from the Bergen adult ADHD study (*N* = 355) and adult blood donors (*N* = 5071) (Supplementary Table 1).

The MoBa study is a nationwide prospective population-based pregnancy cohort that includes 114,500 children, born between 1999 to 2009, and their mothers (fathers also available in the majority of cases). Blood samples were collected from both parents during pregnancy, and from the umbilical cord for the children after birth. For a more detailed description of the sample see Magnus et al.^34,35^. Written informed consent was obtained from all mothers and fathers participating in the study, and the Regional Comittee for Medical Research Ethics (REC) as well as the Norwegian Data Inspectorate approved the MoBa study. For this specific study, a separate REC approval has been obtained. The MoBa data has been linked to the Patient registry to identify ADHD cases. All data have been deidentified prior to analyses.

The Bergen adult ADHD sample consists of participants recruited through a Norwegian national medical registry and by psychologists and psychiatrists at out-patient clinics. ADHD diagnosis was defined according to DSM-IV criteria as described elsewhere^36^. Random controls were recruited through the Norwegian Medical Birth registry. All participants provided either blood or saliva samples for DNA extraction. All participants provided signed informed consent. The study was approved by the Norwegian regional medical research ethics committee West (IRB #3 FWA00009490, IRB00001872).

The Norwegian Blood donors (Oslo University Hospital, Ullevål Hospital, between 18 and 60 years) were included in the control sample. They were all thoroughly screened for diseases, and provided blood for DNA analysis, in line with approval from the Regional Committee for Medical and Health Research Ethics.

### Neuropsychiatric CNV identification and calling

Nineteen CNVs conferring risk of schizophrenia or ASD (‘neuropsychiatric CNVs’) were selected based on recent publications^17–19,37,38,39^ (Supplementary Table 2). Icelandic subjects carrying neuropsychiatric CNVs were identified from a large genotyped sample (*N* = 160,772). The samples were genotyped using the Illumina HumanHap (300, 370, 610, 1M, 2.5M) and IlluminaOmni (670, 1M, 2.5M, Express) SNP arrays. Norwegian neuropsychiatric CNV carriers were identified from 36,220 samples genotyped on Illumina SNP arrays (OmniExpress or Global Screening Array).

Genomestudio (Illumina; version v2011.1) was used to call genotypes, normalize signal intensity data and establish the log R ratio (LRR) and B allele frequency (BAF) for every SNP. PennCNV^40^ was then used to predict CNVs from the SNP array data. Samples with LRR standard deviation over 0.3 or BAF drift over 0.01 were discarded from the analyses. The putative neuropsychiatric CNVs of all samples were confirmed by visual inspection of LRR and BAF plots over each predicted CNV region.

### CNV association

The CNVs under investigation are rare, and in order to estimate the minimum population frequency required to have 80% power to detect an association with an OR of above 3.9, we used the effect size calculation in the chi-squared test for association function (ES.w2) in the basic functions for power analysis (pwr) package (version 1.2–2) in R; pwr.chisq.test, *N* = 163,409 (correction factor (see below) adjusted, Icelandic and Norwegian samples), degrees of freedom = 1, significance level = 0.05.

To evaluate whether the neuropsychiatric CNVs were significantly enriched in our Icelandic and Norwegian ADHD sample, the number of case-carriers, case-noncarriers, control-carriers and control-noncarriers was determined per CNV, and CNVs combined, for each sample and an odds ratio (OR) and *P* value were estimated using a Fisher exact test (fisher.test) in R. To account for relatedness within the Icelandic and Norwegian samples, the *P* values were adjusted with a correction factor (1.187 and 1.033, respectively) estimated using the intercept from LD score regression^41^. Prior to meta-analysis, the Icelandic and Norwegian ADHD affected and control, carrier and non-carrier counts, were adjusted with the above correction factors, rounded to the nearest integer, and then combined using the Cochran–Mantel–Haenszel *χ*^2^ test for count data (mantelhaen.test) in R. We used RStudio (version 1.0.44; https://www.rstudio.com/) integrating R (version 3.3.2; https://www.r-project.org/) and employing the stats base package for the association tests and ggplot2 (version 2.2.1) to generate the power figure.

## Results

Here we meta-analyze ADHD data from Iceland and Norway (*N* = 8883 ADHD affected) (Methods and Supplementary Table 1). The Icelandic sample combines two ADHD study groups, a group of subjects diagnosed with ADHD and a group of subjects assumed to have ADHD based on prescription of ADHD medication. The subjects diagnosed with ADHD are on average 13.6 years younger than those prescribed medication for ADHD (mean age 30.3 and 43.9, respectively), and the combined ADHD sample has a male to female ratio of 3:2. The Norwegian ADHD sample, with a male to female ratio of 2:1, were from the Norwegian mother and child cohort study (MoBa)^34,35^, which includes children and adults, and from the Bergen adult ADHD study^36^.

We compiled a list of 19 neuropsychiatric CNVs that have been shown to confer risk of schizophrenia and/or ASD^17–19,37,38,39^ (Supplementary Table 2). All but the 2p16.3 deletions are recurrent and flanked by segmental duplications (Supplementary Fig. 1). All samples were genotyped using Illumina SNP arrays and the preselected neuropsychiatric CNVs were identified using the PennCNV algorithm and confirmed by visual inspection and segregation in pedigrees (Methods). Individually these CNVs are rare (0.0027–0.25% carrier frequency in the population), and we estimated that at 80% power a CNV with a frequency of 0.018% or greater was required to detect an association with an OR above 3.9 (Supplementary Fig. 2 and Methods). The individual associations were therefore restricted to CNVs with a population frequency of >0.018% in the combined Icelandic and Norwegian sample; deletions at 1q21.1 distal, 2p16.3 (*NRXN1*), 15q11.2, 15q13.3 (break point (BP)4 & BP4.5–BP5), 16p11.2 distal, 16p11.2 proximal, 16p12.1, 17p12 and 22q11.21 and duplications at 1q21.1 distal, 16p11.2 proximal, 16p13.11, 17q12 and 22q11.21.

**Fig. 1:**
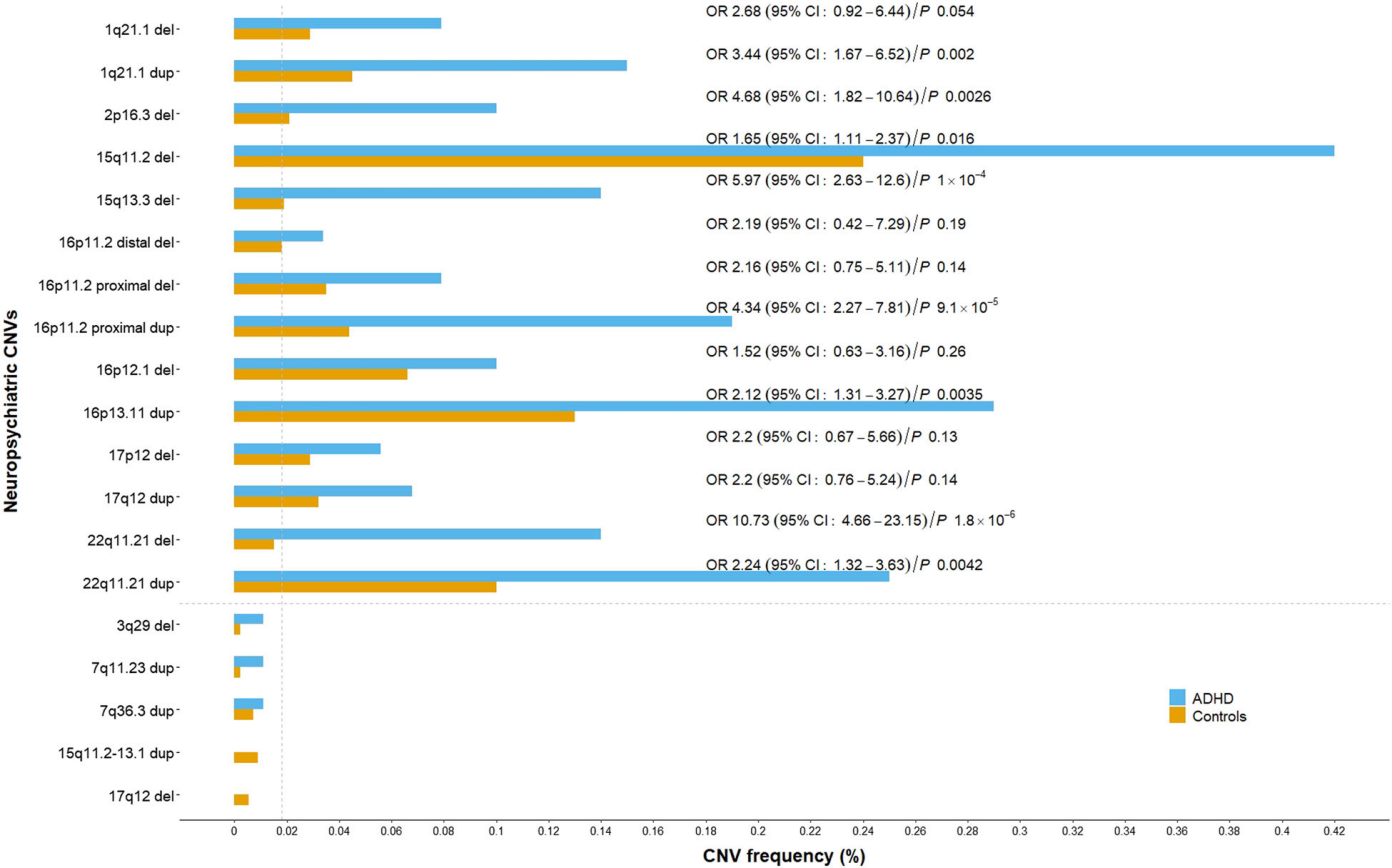
Neuropsychiatric CNV association with ADHD in Icelandic and Norwegian samples. The affected and control carrier frequencies were calculated from the combined number of CNV carriers divided by the number of genotyped individuals in the Icelandic and Norwegian sample combined, before adjusting the counts for relatedness. For Icelandic and Norwegian population frequency separately, see Supplementary Table 3. To estimate OR and *P* values, the Icelandic and Norwegian affected and control, carrier and non-carrier counts, were adjusted for relatedness with a correction factor (1.187 in Iceland and 1.033 in Norway) using the intercept from LD score regression^41^, rounded to the nearest integer, and then combined using the Cochran–Mantel–Haenszel *χ*^2^ test for count data. The *P* values were further adjusted by false discovery rate (FDR). Neuropsychiatric CNVs below the horizontal, dashed, gray line were not tested as their population carrier frequency was below 0.018% (vertical, dashed, gray line) required for the 80% power to detect CNVs with an OR 3.9

Of the 14 CNVs tested, the two previously associated with ADHD^23,26^, 16p13.11 duplication and 22q11.21 deletion were replicated in the combined Icelandic and Norwegian sample (OR (95% CI) = 2.12 (1.31, 3.27), *P* = 0.0035 and OR (95% CI) = 10.73 (4.66, 23.15), *P* = 1.8 × 10^−6^, respectively; Cochran–Mantel–Haenszel *χ*^2^ test for count data and false discovery rate (FDR) adjusted *P* value); it should be noted that a part of the Icelandic sample was included in the original 16p13.11 duplication study^23^ (Fig. 1, Table 1 and Supplementary Table 3).

**Table 1:** Neuropsychiatric CNV association with ADHD in Icelandic and Norwegian samples

Neuropsychiatric CNV loci tested	Iceland and Norway combined	
	Affected/control carrier frequency (%)^a^	OR (95% CI), *P* (FDR adjusted)^b^
1q21.1 distal—deletion	0.0788/0.0288	2.68 (0.92, 6.44), 0.054
1q21.1 distal—duplication	0.146/0.0454	3.44 (1.67, 6.52), 0.0020
2p16.3 (NRXN1)—deletion	0.101/0.0210	4.68 (1.82, 10.64), 0.0026
15q11.2—deletion	0.417/0.244	1.65 (1.11, 2.37), 0.016
15q13.3 (BP4 & BP4.5–BP5) deletion^c^	0.135/0.0194	5.97 (2.63, 12.6), 1.0 × 10^−4^
16p11.2 distal—deletion	0.0338/0.0177	2.19 (0.42, 7.29), 0.19
16p11.2 proximal-deletion	0.0788/0.0354	2.16 (0.75, 5.11), 0.14
16p11.2 proximal—duplication	0.191/0.0437	4.34 (2.27, 7.81), 9.1 × 10^−5^
16p12.1—deletion	0.101/0.0664	1.52 (0.63, 3.16), 0.26
16p13.11—duplication	0.293/0.129	2.12 (1.31, 3.27), 0.0035
17p12—deletion	0.0563/0.0288	2.20 (0.67, 5.66), 0.13
17q12—duplication	0.0675/0.0315	2.20 (0.76, 5.24), 0.14
22q11.21—deletion	0.135/0.0155	10.73 (4.66, 23.15), 1.8 × 10^−6^
22q11.21—duplication	0.248/0.100	2.24 (1.32, 3.63), 0.0042

^a^The affected and control carrier frequencies were calculated from the combined number of CNV carriers divided by the number of genotyped individuals in the Icelandic and Norwegian sample combined, before adjusting the counts for relatedness. For Icelandic and Norwegian population frequency separately, see Supplementary Table 3

^b^The Icelandic and Norwegian affected and control, carrier and non-carrier counts, were adjusted for relatedness with a correction factor (1.187 in Iceland and 1.033 in Norway) using the intercept from LD score regression^41^, rounded to the nearest integer, and then combined using the Cochran–Mantel–Haenszel *χ*^2^ test for count data. The *P* values were further adjusted by false discovery rate (FDR)

^c^BP—break point

Previous reports have shown a higher frequency of ADHD in carriers of six (deletions at 1q21.1 distal, 15q11.2, 15q13.3 (BP4 & BP4.5–BP5) and 16p11.2 proximal and duplications at 1q21.1 distal and 16p11.2 proximal) of the remaining 12 CNVs, although not statistically tested^27–29,32^. We present evidence of significant association with ADHD for deletions at 15q11.2 and 15q13.3 (BP4 & BP4.5–BP5) and duplications at 1q21.1 distal and 16p11.2 proximal. The remaining six CNVs have not been associated with a diagnosis of ADHD before; the deletions at 2p16.3 (*NRXN1*), 16p11.2 distal, 16p12.1 and 17p12 and duplications at 17q12 and 22q11.21. Of those, the 2p16.3 (*NRXN1*) deletion and 22q11.21 duplication were significant in the combined sample (OR (95% CI) = 4.68 (1.82, 10.64), *P* = 0.0020 and OR (95% CI) = 2.24 (1.32, 3.63), *P* = 0.0042, respectively; Cochran–Mantel–Haenszel *χ*^2^ test for count data and FDR adjusted *P* value) (Fig. 1, Table 1 and Supplementary Table 3). Affected and control, carrier frequency for the five remaining, individually untested, CNVs are given in Fig. 1 and Supplementary Table 4.

Combining the 19 neuropsychiatric CNVs, we performed a Cochran–Mantel–Haenszel *χ*^2^ test for count data on the Icelandic and Norwegian ADHD samples to estimate the overall CNV burden. This revealed a significant association with ADHD (OR (95% CI) = 2.43 (2.05, 2.87), *P* = 1.6 × 10^−21^; counts adjusted for correction factor, see Methods). The combined neuropsychiatric CNVs have a carrier frequency of 2.15% in the Icelandic and Norwegian ADHD sample compared with 0.86% in the combined controls. The associations with ADHD in the Icelandic and Norwegian samples, separately, were similar (OR (95% CI) = 2.30 (1.86, 2.80), *P* = 1.4 × 10^−11^ and OR (95% CI) = 2.66 (2.04, 3.43), *P* = 7.7 × 10^−12^, respectively; Fisher’s exact test and corrected *P* values) (Table 2).

**Table 2:** Meta-analysis of combined neuropsychiatric CNV association with ADHD in Icelandic and Norwegian samples

Neuropsychiatric CNVs	Iceland carrier frequency (%)^d^	OR (95% CI), *P* corrected^e^	Norway carrier frequency (%)^d^	OR (95% CI), *P* corrected^e^	Combined carrier frequency (%)^f^	OR (95% CI), *P*^f^
Combined^a^	1.89/0.834	2.30 (1.86, 2.80), 1.4 × 10^−11^	2.54/0.971	2.66 (2.04, 3.43), 7.7 × 10^−12^	2.15/0.855	2.43 (2.05, 2.87), 1.6 × 10^−21^
Combined^b^	0.389/0.235	1.66 (1.03, 2.55), 0.041	0.557/0.230	2.43 (1.35, 4.18), 0.0034	0.456/0.235	1.94 (1.33, 2.76), 6.0 × 10^−4^
Combined^c^	0/0.0264	0 (0, 2.58), 0.44	0.0928/0.0234	3.97 (0.64, 18.59), 0.075	0.0380/0.0257	1.38 (0.27, 4.42), 0.49

^a^All 19 combined: 1q21.1 distal—deletion, 1q21.1 distal—duplication, 2p16.3 (NRXN1)—deletion, 3q29—deletion, 7q11.23 (WBS)—duplication, 7q36.3 (VIPR2)—duplication, 15q11.2—deletion, 15q11.2–13.1—duplication, 15q13.3 (BP4 & BP4.5–BP5)—deletion, 16p11.2 distal—deletion, 16p11.2 proximal—deletion, 16p11.2 proximal—duplication, 16p12.1—deletion, 16p13.11—duplication, 17p12—deletion, 17q12—deletion, 17q12—duplication, 22q11.21—deletion, 22q11.21—duplication

^b^Removed eight individually significant CNVs (Fig. 1, Table 1 and Supplementary Table 3) and tested: 1q21.1 distal—deletion, 3q29—deletion, 7q11.23 (WBS)—duplication, 7q36.3 (VIPR2)—duplication, 15q11.2–13.1—duplication, 16p11.2 distal—deletion, 16p11.2 proximal—deletion, 16p12.1—deletion, 17p12—deletion, 17q12—deletion, 17q12—duplication

^c^Combined individually untested CNVs from Supplementary Table 4: 3q29—deletion, 7q11.23 (WBS)—duplication, 7q36.3 (VIPR2)—duplication, 15q11.2–13.1—duplication, 17q12—deletion

^d^The affected/control carrier frequencies were calculated from the combined number of CNV carriers divided by the combined number of genotyped individuals in the Icelandic and Norwegian sample separately, before adjusting the counts for relatedness. Iceland ADHD affected (*N* = 5650) and controls (*N* = 155,122), and Norwegian ADHD affected (*N* = 3233) and controls (*N* = 25,654)

^e^Odds ratio (OR), 95% confidence interval (95% CI) and *P* value are estimated using Fisher's exact to test for increased burden of the neuropsychiatric CNVs in the ADHD cases compared with controls in the Icelandic or Norwegian samples. The *P* values were adjusted with a correction factor (1.187 in Iceland and 1.033 in Norway) using the intercept from LD score regression^41^

^f^The Icelandic and Norwegian carrier frequency (in percent) was calculated from affected/control, carrier and non-carrier counts, adjusted for relatedness with a correction factor (1.187 in Iceland and 1.033 in Norway) using the intercept from LD score regression^41^, and rounded to the nearest integer, and then to estimate OR combined using the Cochran–Mantel–Haenszel *χ*^2^ test for count data. Iceland and Norwegian combined ADHD affected (*N* = 7890) and controls (*N* = 155,519)

Removing CNVs individually associated with ADHD from the combined set, we found the remaining 11 CNVs (deletions at 1q21.1 distal, 3q29, 16p11.2 distal, 16p11.2 proximal, 16p12.1, 17p12 and 17q12 and duplications at 7q11.23, 7q36.3, 15q11.2–13.1, 17q12) still conferred a significant risk of ADHD (OR (95% CI) = 1.94 (1.33, 2.76), *P* = 6.0 × 10^−4^; Cochran–Mantel–Haenszel *χ*^2^ test for count data in the Icelandic and Norwegian sample counts adjusted with correction factors and combined). Although, this appears to be mostly accounted for by the six, relatively, more common CNVs tested, as when they are removed the OR is no longer significant for the five remaining CNVs (OR (95% CI) = 1.38 (0.27, 4.42), *P* = 0.49; Cochran–Mantel–Haenszel *χ*^2^ test for count data in the Icelandic and Norwegian sample counts adjusted with correction factors and combined) (Table 2).

We also explored what effect removing individuals with a diagnosis of ASD or schizophrenia, from the Icelandic sample of ADHD affected and controls, would have on the risk for ADHD conferred by the neuropsychiatric CNVs, and found a modest weakening of the signifcance as expected but a subtle increase in OR for nine out of 14 CNVs. However, the CNVs combined showed a similar, although less significant, effect (OR (95% CI) = 2.26 (1.81, 2.79), *P* = 1.2 × 10^−11^; Fisher’s exact test) (Supplementary Table 5).

For the Norwegian sample, the issue of ADHD comorbid with axis I to III disorders was addressed in a recent study where the authors found children with ADHD in MoBa were registered with fewer abnormal psychosocial situations (axis I disorders) compared with children in the general population^42^. Within the Bergen sample, none of the CNV carriers has a diagnosis of schizophrenia and only one is comorbid ADHD and ASD. It is, therefore, unlikely that the comorbid ASD or schizophrenia are responsible for the risk conferred by the neuropsychiatric CNVs present in the Norwegian sample.

We note that the OR for the neuropsychiatric CNVs appears to be higher in the sample of Icelandic subjects with an ADHD diagnosis than in the medication sample (OR (95% CI) = 3.49 (2.72, 4.42), *P* = 1.6 × 10^−17^ and OR (95% CI) = 1.25 (0.84, 1.79), *P* = 0.25, respectively; Fisher’s exact test and corrected *P* values. *P* value for difference = 2.4 × 10^−5^) (Supplementary Tables 6 and 7). A corresponding analysis in the Norwegian sample reveals a comparable, although nonsignificant (*P* = 0.10), trend where the OR is higher in children with ADHD than in adults with ADHD (OR (95% CI) = 2.69 (1.89, 3.78), *P* = 5.9 × 10^−8^ and OR (95% CI) = 1.56 (0.86, 2.63), *P* = 0.11, respectively; Fisher’s exact test and corrected *P* value) (Supplementary Tables 8 and 9).

## Discussion

The results of the current study, combining large ADHD samples from relatively homogenous populations, in terms of genetics and health care systems, support previous findings of increased burden of rare CNVs among ADHD patients^23^. The neuropsychiatric CNVs confer a substantial risk of ADHD in the Icelandic and Norwegian samples. As the CNVs are individually rare, we estimated that 14 were powered to detect a significant association. When we looked at carrier status of the neuropsychiatric CNVs separately, eight were significantly associated with ADHD risk after adjusting for FDR: deletions at 2p16.3 (*NRXN1*), 15q11.2, 15q13.3 (BP4 & BP4.5–BP5) and 22q11.21, and duplications at 1q21.1 distal, 16p11.2 proximal, 16p13.11 and 22q11.21. Two of these CNVs have previously been associated with ADHD, the 16p13.11 duplication and 22q11.21 deletion. Of the remaining six, four have been previously reported with higher frequency of ADHD in carriers but not statistically tested and the deletion at 2p16.3 spanning exons of *NRXN1* and the 22q11.21 duplication have not been associated with ADHD diagnosis before.

The CNV conferring the highest risk of ADHD in our study is the deletion at 22q11.21. The 22q11.2 deletion syndrome (DiGeorge Syndrome) is associated with high rates of schizophrenia spectrum disorders and has been exploited as a genetic model for understanding the development of schizophrenia^26^. Of other psychiatric conditions associated with the deletion, ADHD has been shown to be the most frequent disorder in children (37%) with the inattentive presentation persisting into adulthood^26^.

The 16p13.11 duplication, which has been associated with schizophrenia^19^ and ADHD^23,43,44^, also reached significance threshold in this study, although it should be noted that a part of the Icelandic sample was included in the original Williams et al. study^23^.

The 1q21.1 distal CNV has been associated with multiple phenotypes^27^, including neurodevelopmental and psychiatric disorders, deletions more strongly with schizophrenia^19^ and duplications with ASD^17^. An increase in the frequency of ADHD among both 1q21.1 distal deletion (5%) and duplication (29%) carriers has also been reported^27^. While we confirm this observation with an association of the 1q21.1 distal duplication with ADHD, the 1q21.1 distal deletion is not significant.

The 15q11.2 deletion has been associated with schizophrenia^19^ but also with learning difficulties and brain structural changes^20,45^, as well as an increased frequency of ADHD in carriers^28^. We see a modest association with ADHD in the combined sample.

The 15q13.3 (BP4 & BP4.5–BP5) deletion has previously been associated with mental retardation, seizures, dysmorphic features and schizophrenia^46^. A review of 15q13.3 deletions, involving 246 cases with deletions overlapping the 15q13.3 (BP4–BP5) region, found an increased frequency of neuropsychiatric conditions, including ADHD (6.5%)^29^. We see a highly significant association with ADHD, marginally stronger in the Norwegian sample.

The proximal duplication at 16p11.2 has been associated with schizophrenia^19^ and ASD^17^ but also reported a higher frequency (39–60%) of ADHD in carriers^32,47^. We report a strong and highly significant association with ADHD in our combined sample. 16p11.2 proximal deletion, although not significantly associated with ADHD, showed a definite reversal of OR after excluding individuals with a diagnosis of ASD or schizophrenia from the ADHD and controls samples.

Deletions spanning exons of *NRXN1* have been identified in individuals diagnosed with a range of neurodevelopmental disorders, including intellectual disability, speech and language delay, ASD and schizophrenia^48^ but to our knowledge not ADHD, apart from two clinical referrals for diagnostic cytogenetic analysis^49^. We observe a modest association between deletions removing *NRXN1* exons at 2p16.3 and ADHD in our study.

Current understanding of the 22q11.21 duplication clinical phenotype is quite diverse, but range from ASD^17^, severe mental retardation, dysmorphic facial features and heart malformations^38^ to no signs at all. Notably, while all of the neuropsychiatric CNVs have been associated with increased risk of either schizophrenia or ASD, it has been postulated that the 22q11.21 duplication may confer protection against schizophrenia^37,39,50^. The 22q11.21 duplication has not been previously associated with ADHD in a population sample, although a Danish nationwide CNV registry study did find a modest increase in “any psychiatric disorder” (including ADHD) diagnosis in 22q11.2 duplication carriers^51^. Furthermore, a prospective study of a cohort of children, found ADHD symptom scores to be significantly higher in 22q11.2 duplication carriers in comparison to typically developing children^38^. Only the 22q11.21 CNV has been shown to have a mirror effect on a psychiatric disorder: The deletion confers risk of schizophrenia^26^ whereas the reciprocal duplication has been postulated to be protective against the same disorder^37^. Interestingly, here we demonstrate that both alleles of the 22q11.2 CNV confer risk of ADHD.

Although only a subset of the neuropsychiatric CNVs were predicted to provide well-powered estimates of ADHD risk, all 19 combined were, unsurprisingly, highly significantly associated with ADHD. To explore whether any of the individually non-significant or untested CNVs conferred some latent risk of ADHD, we combined them in two seperate sets. While the slightly more common, but individually non-significant, CNVs combined did reveal an association with ADHD, the smaller set of very rare CNVs combined were not significant. A larger sample of carriers is required to ascertain whether those CNVs associate with ADHD.

A potential confounder of analyses such as the one presented here is the presence of ASD or schizophrenia comorbid with ADHD and the risk conferred by the neuropsychiatric CNVs being attributable to those conditions. We reanalyzed the individual and combined neuropsychiatric CNVs, after excluding individuals with a diagnosis of ASD or schizophrenia in both the ADHD and control samples and found only modest changes in the risk estimates and as expected some change in the *P* values. However, the overall conclusion is that these CNVs, individually or combined, confer risk of ADHD with or without ASD or schizophrenia.

As noted, neuropsychiatric CNVs appear to confer greater risk of ADHD in individuals with a diagnosis compared with those prescribed medication for ADHD. The sample with a diagnosis is younger than those on medication and so rather than an actual difference in risk between the two groups this, more likely, reflects a more complete recruitment for the younger sample and so a broader spectrum of ADHD risk variants are represented whereas high risk CNVs are likely to be underrepresented in the older medication sample. With the proviso that diagnostic criteria and clinical practice are stable over time, it is likely that if individuals born prior to the commencement of systematic ADHD screening and diagnosis would be better represented in ADHD study samples, an increase in CNV frequency would also be observed.

In this study, we show that neuropsychiatric CNVs, previously associated with schizophrenia and ASD, also confer risk of ADHD. Hence, further emphasizing the pleiotropic effects of CNVs. This adds to the evidence that these disorders are related rather than etiologically distinct entities and supports previous findings of both common and rare variant sharing. While, a unifying factor of the neuropsychiatric CNVs is their negative impact on cognitive abilities and disadvantages in educational attainment that potentially explain part of their association with psychiatric disorders, environmental interactions and other sequence variants in CNV carriers may affect the disorder expressed.

## Supplementary information

Supplementary Information
