# Supplementary material for: Attention-deficit hyperactivity disorder shares copy number variant risk with schizophrenia and autism spectrum disorder
Source: Transl Psychiatry. 2019 Oct 17;9:258. doi: 10.1038/s41398-019-0599-y (PMC6797719; doi:10.1038/s41398-019-0599-y)
Supplement: Supplementary file 1 — Supplementary Information [file 41398_2019_599_MOESM1_ESM.docx]

Attention deficit-hyperactivity disorder shares copy number variant risk with schizophrenia and autism spectrum disorder

**Supplementary Information**

**Supplementary Tables**

Supplementary Table 1. Demographics for samples from Iceland and Norway.

Supplementary Table 2. Neuropsychiatric CNVs.

Supplementary Table 3. Neuropsychiatric CNV association and meta-analysis with ADHD in Icelandic and Norwegian samples.

Supplementary Table 4. Carrier frequency of neuropsychiatric CNVs not tested for association with ADHD in Icelandic and Norwegian samples.

Supplementary Table 5. Individual neuropsychiatric CNV ADHD association in the Icelandic sample excluding individuals with a diagnosis of autism spectrum disorder or schizophrenia.

Supplementary Table 6. Neuropsychiatric CNV ADHD association in the Icelandic sample subgroups.

Supplementary Table 7. Individual neuropsychiatric CNV ADHD association in the Icelandic ADHD diagnosed or medication sample subgroups.

Supplementary Table 8. Neuropsychiatric CNV ADHD association in the Norwegian sample subgroups.

Supplementary Table 9. Individual neuropsychiatric CNV ADHD association in the Norwegian sample subgroups.

**Supplementary Figures**

Supplementary Figure 1. Neuropsychiatric CNVs found in Icelandic and Norwegian ADHD cases or controls. Chromosome, start and stop in human genome build 18 (hg18-NCBI36).

Supplementary Figure 2. Estimate of minimun CNV population frequency required to detect association.

**References**

**SUPPLEMENTARY TABLES**

**Supplementary Table 1. Demographics for samples from Iceland and Norway.**

|  |  | **Male** | | **Female** | |
| --- | --- | --- | --- | --- | --- |
| **Sample** | **Total** | **count** | **mean YOB** | **count** | **mean YOB** |
| **Iceland** | |  |  |  |  |
| **ADHD diagnosis** | 2 665 | 1 746 | 1990.1 | 919 | 1984.3 |
| **ADHD medication** | 2 985 | 1 489 | 1974.8 | 1 496 | 1973.1 |
| **Controls** | 155 122 | 71 492 | 1958.2 | 83 630 | 1958.3 |
| **Norway** | |  |  |  |  |
| **ADHD – children (MoBa)** | 1 858 | 1 340 | 2003.9 | 518 | 2004.0 |
| **ADHD - adults (MoBa)** | 941 | 327 | 1975.0 | 614 | 1977.2 |
| **ADHD – adults (Bergen)** | 434 | 204 | na | 230 | na |
| **Controls - children** | 8 245 (2 951) | 4 552 | 2005.4 | 3 693 | 2005.7 |
| **Controls - adults (MoBa)** | 19 316 (17 277) | 9 162 | 1972.6 | 10 154 | 1975.1 |
| **Controls - adults (Blood donors)** | 5 071 | 2 654 | na | 2 417 | na |
| **Controls - adults (Bergen)** | 355 | 146 | na | 209 | na |

YOB - year of birth. MoBa - Mor & Barn (Mother and child). Blood Donors (BD) are required to be above 18 before donating blood. Norwegian adult controls are made up of MoBa, BD and Bergen samples. The values within parentheses in the Norwegian control sample are the number of individuals used in the combined adult and child analysis, after removing the unaffected parents of affected children and the unaffected children of unaffected parents from control list.

**Supplementary Table 2. Neuropsychiatric CNVs.**

| **Neuropsychiatric CNVs ^1^** | **Start ^2^** | **Stop ^2^** | **OR, *P* (SCH / ASD)** | **References** |
| --- | --- | --- | --- | --- |
| 1q21.1 distal - deletion | 144 967 972 | 146 292 172 | 8.35, 4.1×10^-13^ / - | SCH ^1, 2^ |
| 1q21.1 distal - duplication | 144 967 972 | 146 292 172 | 3.45, 9.9×10^-5^ / 8, 3.6×10^-5^ | SCH ^1, 2^; ASD ^1^ |
| 2p16.3 (NRXN1) - deletion | 49 999 147 | 51 113 178 | 9.01, 1.3×10^-11^ / 5.6, 0.0086 | SCH^2^; ASD ^3^ |
| 3q29 - deletion | 197 251 134 | 198 700 746 | 57.65, 1.9×10^-9^ / - | SCH^1, 2^ |
| 7q11.23 (WBS) - duplication | 72 360 917 | 73 793 214 | 11.35, 6.6×10^-5^ / 30.7, 8.0×10^-4^ | SCH^1, 2^; ASD^1^ |
| 7q36.3 (VIPR2) - duplication | 158 419 223 | 158 640 055 | 3.2, 0.006 / - | SCH^1^ |
| 15q11.2 - deletion | 20 301 669 | 20 824 174 | 2.15, 2.5×10^-10^ / - | SCH^1, 2^ |
| 15q11.2-13.1 (BP1-2) - duplication | 20 322 358 | 26 208 861 | 13.2, 5.6×10^-6^ / 42.6, 1.1×10^-15^ | SCH^1, 2^; ASD^1, 3^ |
| 15q13.3 (BP4 & BP4.5 - BP5) – deletion ^3^ | 28 723 577 (29 806 023) | 30 303 141 | 7.52, 4×10^-10^ / 10.8, 0.001 | SCH^1, 2^; ASD^1, 3^ |
| 16p11.2 distal - deletion | 28 710 000 | 28 955 000 | 20.6, 5.5×10^-5^ / - | SCH^4^ |
| 16p11.2 proximal - deletion | 29 502 984 | 30 100 062 | - / 9.5, 2.0×10^-10^ | ASD^1, 3^ |
| 16p11.2 proximal - duplication | 29 502 984 | 30 105 652 | 11.52, 2.9×10^-24^ / 11.8, 6.2×10^-11^ | SCH^1, 2^; ASD^1^ |
| 16p12.1 - deletion | 21 854 731 | 22 331 199 | 2.72, 0.0012 / - | SCH^2^ |
| 16p13.11 - duplication | 15 400 547 | 16 199 484 | 2.3, 5.7×10^-5^ / - | SCH^1, 2^ |
| 17p12 - deletion | 14 041 754 | 15 411 904 | 3.62, 0.0012 / - | SCH^1^ |
| 17q12 - deletion | 31 889 664 | 33 323 543 | 6.64, 0.0072 / 16, 0.01 | SCH^1, 2^; ASD^1^ |
| 17q12 - duplication | 31 889 664 | 33 323 543 | 4.94, 0.0095 / - | SCH^2^ |
| 22q11.21 – deletion ^4^ | 17 266 915 | 19 79 3370 | inf, 4.4×10^-40^ / inf, 0.002 | SCH^1, 2^; ASD^1^ |
| 22q11.21 – duplication ^4^ | 17 266 915 | 19 793 370 | 0.17, 8.6×10^-4^ / 3.3, 0.002 | SCH^5^; ASD^1, 6^ |

ASD - Autism spectrum disorder, BP – Break-point, SCH - Schizophrenia, WBS – William Beuren Syndrome locus.

^1^ The start and stop of these CNVs represent the core regions tested per locus; although two or more variably sized fragments are observed. Each carrier subject is required to have a CNV spanning this interval in order to be included in the analyses.

^2^ Positions are in human genome build 18 (hg18 - NCBI36).

^3^ The number outside parentheses refers to break-point (BP) 4, while within parentheses refers to BP4.5, found at the 15q13.3 locus.

^4^ There are multiple breakpoints for both 22q11.21 deletions and duplications and, including the ones in the table, are 17 292 678-18 666 099, 19 063 495-19 793 370 and 19 424 781-19 793 370.

**Supplementary Table 3. Neuropsychiatric CNV association and meta-analysis with ADHD in Icelandic and Norwegian samples.**

| **Neuropsychiatric CNV Loci tested** | **ICELAND** | | **NORWAY** | | **ICELAND AND NORWAY COMBINED** | | |
| --- | --- | --- | --- | --- | --- | --- | --- |
|  | **affected / control carrier frequency (%) ^1^** | **OR (95% CI),**  ***P* corrected ^2^** | **affected / control carrier frequency (%) ^1^** | **OR (95% CI),**  ***P* corrected ^2^** | **affected / control carrier frequency (%) ^3^** | **population frequency (%) ^3^** | **OR (95% CI),**  ***P, P* (FDR adjusted) ^4^** |
| 1q21.1 - distal - deletion | 0.0708 / 0.0297 | 2.39 (0.62, 6.54), 0.13 | 0.0928 / 0.0234 | 3.97 (0.64, 18.59), 0.075 | 0.0788 / 0.0288 | 0.0311 | 2.68 (0.92, 6.44), 0.035, 0.054 |
| 1q21.1 - distal - duplication | 0.0885 / 0.0483 | 1.83 (0.58, 4.47), 0.25 | 0.247 / 0.0273 | 9.09 (2.88, 29.46), 9.9 × 10^-5^ | 0.146 / 0.0454 | 0.0501 | 3.44 (1.67, 6.52), 5.8 × 10^-4^, 0.0020 |
| 2p16.3 (NRXN1) - deletion | 0.124 / 0.0200 | 6.21 (2.31, 14.37), 9.6 × 10^-4^ | 0.0619 / 0.0273 | 2.27 (0.23, 11.92), 0.28 | 0.101 / 0.0210 | 0.0249 | 4.68 (1.82, 10.64), 9.3 × 10^-4^, 0.0026 |
| 15q11.2 - deletion | 0.442 / 0.237 | 1.87 (1.19, 2.81), 0.011 | 0.371 / 0.285 | 1.31 (0.64, 2.42), 0.4 | 0.417 / 0.244 | 0.253 | 1.65 (1.11, 2.37), 0.0089, 0.016 |
| 15q13.3 (BP4 & BP4.5 - BP5) - deletion | 0.0885 / 0.0181 | 4.91 (1.48, 12.87), 0.011 | 0.217 / 0.0273 | 7.95 (2.38, 26.58), 4.6 × 10^-4^ | 0.135 / 0.0194 | 0.0253 | 5.97 (2.63, 12.6), 2.2 × 10^-5^, 1.0 × 10^-4^ |
| 16p11.2 distal - deletion | 0.0354 / 0.0181 | 1.96 (0.23, 7.78), 0.33 | 0.0309 / 0.0156 | 1.98 (0.04, 20.06), 0.46 | 0.0338 / 0.0177 | 0.0184 | 2.19 (0.42, 7.29), 0.18, 0.19 |
| 16p11.2 proximal - deletion | 0.0708 / 0.0361 | 1.96 (0.52, 5.31), 0.2 | 0.0928 / 0.0312 | 2.98 (0.51, 12.42), 0.13 | 0.0788 / 0.0354 | 0.0374 | 2.16 (0.75, 5.11), 0.12, 0.14 |
| 16p11.2 proximal - duplication | 0.177 / 0.0445 | 3.98 (1.83, 7.78), 0.0013 | 0.217 / 0.0390 | 5.56 (1.8, 16.2), 0.0018 | 0.191 / 0.0437 | 0.0508 | 4.34 (2.27, 7.81), 1.3 × 10^-5^, 9.1 × 10^-5^ |
| 16p12.1 - deletion | 0.106 / 0.0658 | 1.62 (0.58, 3.64), 0.32 | 0.0928 / 0.0702 | 1.32 (0.25, 4.54), 0.72 | 0.101 / 0.0664 | 0.0681 | 1.52 (0.63, 3.16), 0.26, 0.26 |
| 16p13.11 - duplication | 0.230 / 0.122 | 1.88 (0.98, 3.30), 0.052 | 0.402 / 0.172 | 2.35 (1.16, 4.45), 0.011 | 0.293 / 0.129 | 0.138 | 2.12 (1.31, 3.27), 0.0015, 0.0035 |
| 17p12 - deletion | 0.0531 / 0.0290 | 1.83 (0.36, 5.71), 0.28 | 0.0619 / 0.0273 | 2.27 (0.23, 11.92), 0.28 | 0.0563 / 0.0288 | 0.0301 | 2.2 (0.67, 5.66), 0.095, 0.13 |
| 17q12 - duplication | 0.0531 / 0.0303 | 1.75 (0.35, 5.45), 0.30 | 0.0928 / 0.0390 | 2.38 (0.42, 9.25), 0.18 | 0.0675 / 0.0315 | 0.0335 | 2.2 (0.76, 5.24), 0.12, 0.14 |
| 22q11.21 - deletion | 0.142 / 0.0181 | 7.86 (3.09, 17.7), 1.2 × 10^-4^ | 0.124 / 0 | Inf (5.24, Inf), 2.0 × 10^-4^ | 0.135 / 0.0155 | 0.0209 | 10.73 (4.66, 23.15), 1.3 × 10^-7^, 1.8 × 10^-6^ |
| 22q11.21 - duplication | 0.212 / 0.0928 | 2.29 (1.16, 4.13), 0.023 | 0.309 / 0.144 | 2.15 (0.95, 4.41), 0.04 | 0.248 / 0.100 | 0.108 | 2.24 (1.32, 3.63), 0.0021, 0.0042 |

**^1^** The affected and control carrier frequencies were calculated from the combined number of CNV carriers divided by the number of genotyped individuals in the Icelandic and Norwegian samples separately, before adjusting the counts for relatedness.

**^2^** Odds ratio (OR), 95% confidence interval (95% CI) and P-value are estimated using Fisher exact to test for increased burden of the neuropsychiatric CNV in the ADHD cases compared with controls in the Icelandic (5 650 ADHD case and 155 122 control) or Norwegian (3 233 ADHD case and 25 654 control) samples. The P values were adjusted with a correction factor (1.187 in Iceland and 1.033 in Norway) using the intercept from LD score regression^7^.

**^3^** The population frequency was calculated from the combined number of CNV carriers divided by the number of genotyped individuals in the Icelandic and Norwegian samples combined, before adjusting the counts for relatedness.

**^4^** The Icelandic and Norwegian affected and control, carrier and non-carrier counts, were adjusted for relatedness with a correction factor (1.187 in Iceland and 1.033 in Norway) using the intercept from LD score regression^7^, rounded to the nearest integer, and then combined using the Cochran-Mantel-Haenszel Chi-Squared Test for Count Data.

BP – Break-point, FDR - False Discovery Rate.

**Supplementary Table 4. Carrier frequency of neuropsychiatric CNVs not tested for association with ADHD in Icelandic and Norwegian samples.**

| **Neuropsychiatric CNV Loci not tested** | **ICELAND ^1^** | **NORWAY ^1^** | **ICELAND AND NORWAY COMBINED** | |
| --- | --- | --- | --- | --- |
|  | **affected / control carrier frequency (%)** | **affected / control carrier frequency (%)** | **affected / control carrier frequency (%) ^2^** | **population frequency (%) ^3^** |
| 3q29 - deletion | 0 / 0.00258 | 0.0309 / 0 | 0.0113 / 0.00221 | 0.00264 |
| 7q11.23 (WBS) - duplication | 0 / 0.00193 | 0.0309 / 0.00390 | 0.0113 / 0.00221 | 0.00264 |
| 7q36.3 (VIPR2) - duplication | 0 / 0.00516 | 0.0309 / 0.0195 | 0.0113 / 0.00719 | 0.00738 |
| 15q11.2-13.1 - duplication | 0 / 0.0103 | 0 / 0 | 0 / 0.00885 | 0.00844 |
| 17q12 - deletion | 0 / 0.00645 | 0 / 0 | 0 / 0.00553 | 0.00527 |

**^1^** The affected and control carrier frequencies were calculated from the combined number of CNV carriers divided by the number of genotyped individuals in the Icelandic (5 650 ADHD case and 155 122 control) or Norwegian (3 233 ADHD case and 25 654 control) samples seperately, before adjusting the counts for relatedness.

**^2^** The affected and control carrier frequencies were calculated from the combined number of CNV carriers divided by the number of genotyped individuals in the Icelandic and Norwegian samples combined, before adjusting the counts for relatedness.

**^3^** The population frequency was calculated from the combined number of CNV carriers divided by the number of genotyped individuals in the Icelandic and Norwegian samples combined, before adjusting the counts for relatedness.

From power estimates, only CNVs with a population frequency of 0.018% or greater, in the combined sample, were tested individually for association with ADHD (**Figure 1, Table 1** and **Supplementary Table 3**). WBS – Williams-Beuren Syndrome locus.

**Supplementary Table 5. Individual neuropsychiatric CNV ADHD association in the Icelandic sample excluding individuals with a diagnosis of autism spectrum disorder or schizophrenia.**

| **Locus** | **ADHD affected / control carrier frequency (%) ^1^** | **OR (95% CI), *P* corrected ^2^** |
| --- | --- | --- |
| 1q21.1 - distal - deletion | 0.0776 / 0.0293 | 2.65 (0.69, 7.28), 0.10 |
| 1q21.1 - distal - duplication | 0.0970 / 0.0475 | 2.04 (0.64, 4.99), 0.14 |
| 2p16.3 (NRXN1) - deletion | 0.116 / 0.0189 | 6.18 (2.10, 15.14), 2.2 × 10^-3^ |
| 15q11.2 - deletion | 0.466 / 0.235 | 1.99 (1.26, 3.01), 0.0051 |
| 15q13.3 (BP4 & BP4.5 - BP5) - deletion | 0.0970 / 0.0169 | 5.74 (1.72, 15.19), 0.0065 |
| 16p11.2 distal - deletion | 0.0388 / 0.0176 | 2.21 (0.25, 8.80), 0.28 |
| 16p11.2 proximal - deletion | 0.0194 / 0.0332 | 0.59 (0.015, 3.41), 1 |
| 16p11.2 proximal - duplication | 0.136 / 0.0429 | 3.17 (1.22, 6.9), 0.018 |
| 16p12.1 - deletion | 0.0970 / 0.0664 | 1.46 (0.46, 3.53), 0.44 |
| 16p13.11 - duplication | 0.233 / 0.121 | 1.93 (0.98, 3.45), 0.059 |
| 17p12 - deletion | 0.0582 / 0.0286 | 2.03 (0.40, 6.35), 0.24 |
| 17q12 - duplication | 0.0582 / 0.0306 | 1.90 (0.38, 5.92), 0.26 |
| 22q11.21 - deletion | 0.116 / 0.0163 | 7.16 (2.40, 17.91), 1.2 × 10^-3^ |
| 22q11.21 - duplication | 0.213 / 0.0924 | 2.31 (1.13, 4.27), 0.030 |
| **Combined neuropsychiatric CNVs** | **1.82 / 0.815** | 2.26 (1.81, 2.79), 4.9 × 10^-10^ |

**^1^** The carrier frequency was calculated from the combined number of CNV carriers divided by the number of genotyped individuals in the respective Icelandic ADHD and control sample, before adjusting the counts for relatedness. Icelandic ADHD cases (N = 5 153) and controls (N = 153 681), after removing individuals with a diagnosis of autism spectrum disorder or schizophrenia from both samples.

**^2^** Odds ratio (OR), 95% confidence interval (95% CI) and P-value are estimated using Fisher exact to test for increased burden of the neuropsychiatric CNVs in the ADHD cases compared with controls in the Icelandic samples. The P values were adjusted with a correction factor (1.187) using the intercept from LD score regression^7^. For comparison, refer to the Iceland OR column in **Supplementary Table 3**.

**Supplementary Table 6. Neuropsychiatric CNV ADHD association in the Icelandic sample subgroups.**

| **Sample ^1^** | **affected carriers / affected noncarriers / control carriers / control noncarriers** | **OR (95% CI) ^2^** | ***P* corrected ^2^** |
| --- | --- | --- | --- |
| ADHD | 107 / 5 543 / 1 293 / 153 829 | 2.30 (1.86, 2.8) | 1.4 × 10^-11^ |
| ADHD - female | 48 / 2 367 / 710 / 82 920 | 2.37 (1.72, 3.18) | 1.4 × 10^-6^ |
| ADHD - male | 59 / 3 176 / 583 / 70 909 | 2.26 (1.69, 2.96) | 2.6 × 10^-7^ |
| ADHD - Diagnosis | 76 / 2 589 / 1 293 / 153 829 | 3.49 (2.72, 4.42) | 3.6 × 10^-16^ |
| ADHD - Diagnosis - female | 29 / 890 / 710 / 82 920 | 3.81 (2.51, 5.55) | 8.1 × 10^-8^ |
| ADHD - Diagnosis - male | 47 / 1 699 / 583 / 70 909 | 3.37 (2.43, 4.55) | 4.5 × 10^-10^ |
| ADHD - Medication | 31 / 2 954 / 1 293 / 153 829 | 1.25 (0.84, 1.79) | 0.26 |
| ADHD - Medication - female | 19 / 1 477 / 710 / 82 920 | 1.50 (0.9, 2.37) | 0.12 |
| ADHD - Medication - male | 12 / 1 477 / 583 / 70 909 | 0.99 (0.51, 1.74) | 1 |

^1^ The sample contains individuals diagnosed with ADHD according to ICD10 criteria as well as those prescribed medication for ADHD symptoms.

^2^ Odds ratio (OR), 95% confidence interval (95% CI) and P-value are estimated using Fisher exact to test for increased burden of the neuropsychiatric CNVs in the ADHD cases compared with controls in the Icelandic samples. The P values were adjusted with a correction factor (1.187) using the intercept from LD score regression^7^.

**Supplementary Table 7. Individual neuropsychiatric CNV ADHD association in the Icelandic ADHD diagnosed or medication sample subgroups.**

| **Locus** | **ADHD diagnosed carrier frequency (%) ^1^** | **OR (95% CI),**  ***P* (uncorrected) ^2^** | **ADHD medication carrier frequency (%) ^1^** | **OR (95% CI),**  ***P* (uncorrected) ^2^** | **Control carrier frequency (%)** |
| --- | --- | --- | --- | --- | --- |
| 1q21.1 - distal - deletion | 0.0375 | 1.27 (0.031, 7.42), 0.58 | 0.101 | 3.39 (0.67, 10.56), 0.090 | 0.0297 |
| 1q21.1 - distal - duplication | 0.0750 | 1.55 (0.18, 5.81), 0.41 | 0.101 | 2.08 (0.42, 6.32), 0.22 | 0.0483 |
| 2p16.3 (NRXN1) - deletion | 0.263 | 13.18 (4.89, 30.52), 1.9 × 10^-5^ | 0 | 0 (0, 6.57), 1 | 0.0200 |
| 15q11.2 - deletion | 0.750 | 3.18 (1.92, 4.99), 6.7 × 10^-5^ | 0.168 | 0.71 (0.23, 1.66), 0.60 | 0.237 |
| 15q13.3 (BP4 & BP4.5 - BP5) - deletion | 0.150 | 8.33 (2.12, 23.84), 0.0046 | 0.0335 | 1.86 (0.045, 11.23), 0.46 | 0.0181 |
| 16p11.2 distal - deletion | 0.0375 | 2.08 (0.051, 12.59), 0.43 | 0.0335 | 1.86 (0.045, 11.23), 0.46 | 0.0181 |
| 16p11.2 proximal - deletion | 0.113 | 3.12 (0.62, 9.61), 0.11 | 0.0335 | 0.93 (0.023, 5.39), 1 | 0.0361 |
| 16p11.2 proximal - duplication | 0.225 | 5.07 (1.80, 11.62), 0.004 | 0.134 | 3.02 (0.80, 8.08), 0.072 | 0.0445 |
| 16p12.1 - deletion | 0.150 | 2.29 (0.61, 6.04), 0.14 | 0.0670 | 1.02 (0.12, 3.78), 0.74 | 0.0658 |
| 16p13.11 - duplication | 0.375 | 3.07 (1.45, 5.78), 0.0051 | 0.101 | 0.82 (0.17, 2.44), 1 | 0.122 |
| 17p12 - deletion | 0.0375 | 1.29 (0.032, 7.59), 0.57 | 0.0670 | 2.31 (0.27, 8.85), 0.26 | 0.0290 |
| 17q12 - duplication | 0.0750 | 2.48 (0.29, 9.47), 0.24 | 0.0335 | 1.11 (0.027, 6.47), 0.63 | 0.0303 |
| 22q11.21 - deletion | 0.225 | 12.5 (4.23, 30.79), 9.4 × 10^-5^ | 0.0670 | 3.71 (0.43, 14.74), 0.14 | 0.0181 |
| 22q11.21 - duplication | 0.338 | 3.65 (1.63, 7.13), 0.0032 | 0.101 | 1.08 (0.22, 3.23), 0.78 | 0.0928 |
| **Combined neuropsychiatric CNVs** | **2.85** | **3.49 (2.72, 4.42), 3.6 × 10^-16^** | **1.04** | **1.25 (0.84, 1.79), 0.26** | **0.834** |

**^1^** The carrier frequency was calculated from the combined number of CNV carriers divided by the number of genotyped individuals in the respective Icelandic sample subgroups, before adjusting the counts for relatedness. Icelandic ADHD diagnosed sample (N = 2 665), ADHD medication sample (N = 2 985) and control sample (N = 155 122).

**^2^** Odds ratio (OR), 95% confidence interval (95% CI) and P-value are estimated using Fisher exact to test for increased burden of the neuropsychiatric CNVs in the ADHD cases compared with controls in the Icelandic samples. The P values were adjusted with a correction factor (1.187) using the intercept from LD score regression^7^.

**Supplementary Table 8. Neuropsychiatric CNV ADHD association in the Norwegian sample subgroups.**

| **Sample** | **affected carriers / affected noncarriers / control carriers / control noncarriers** | **affected / control**  **carrier frequency (%) ^1^** | **OR (95% CI) ^2^** | ***P* corrected ^2^** |
| --- | --- | --- | --- | --- |
| ADHD – adults and children **^3^** | 82 / 3 151 / 249 / 25 405 | 2.54 / 0.971 | 2.66 (2.04, 3.43) | 7.7 × 10^-12^ |
| ADHD - female | 36 / 1 326 / 118 / 12 404 | 2.64 / 0.942 | 2.85 (1.90, 4.20) | 1.0 × 10^-6^ |
| ADHD - male | 46 / 1 825 / 131 / 13 001 | 2.46 / 0.998 | 2.50 (1.74, 3.54) | 1.3 × 10-^6^ |
| ADHD (MoBa) - children | 57 / 1 801 / 96 / 8 149 | 3.07 / 1.16 | 2.69 (1.89, 3.78) | 5.9 × 10^-8^ |
| ADHD (MoBa) - children - female | 20 / 498 / 43 / 3 650 | 3.86 / 1.16 | 3.41 (1.88, 5.98) | 4.7 × 10^-5^ |
| ADHD (MoBa) - children - male | 37 / 1 303 / 53 / 4 499 | 2.76 / 1.16 | 2.41 (1.53, 3.76) | 1.4 × 10^-4^ |
| ADHD (MoBa) - adults **^4^** | 15 / 926 / 251 / 24 136 | 1.59 / 1.03 | 1.56 (0.86, 2.63) | 0.11 |
| ADHD (MoBa) - adults - female | 13 / 601 / 130 / 12 441 | 2.12 / 1.03 | 2.07 (1.07, 3.69) | 0.027 |
| ADHD (MoBa) - adults - male | 2 / 325 / 121 / 11 695 | 0.612 / 1.02 | 0.60 (0.071, 2.21) | 0.78 |
| ADHD (Bergen) - adults | 10 / 424 / 1 / 354 | 2.30 / 0.282 | 8.33 (1.18, 362.80) | 0.017 |
| ADHD (Bergen) - adults - female | 3 / 227 / 0 / 209 | 1.30 / 0 | Inf (0.38, Inf) | 0.26 |
| ADHD (Bergen) - adults - male | 7 / 197 / 1 / 145 | 3.43 / 0.685 | 5.13 (0.65, 233.5) | 0.15 |

**^1^** The carrier frequency was calculated from the combined number of CNV carriers divided by the number of genotyped individuals in the respective Norwegian ADHD and control sample, before adjusting the counts for relatedness.

**^2^** Odds ratio (OR), 95% confidence interval (95% CI) and P-value are estimated using Fisher exact to test for increased burden of the neuropsychiatric CNVs in the ADHD cases compared with controls in the Norwegian samples. The P values were adjusted with a correction factor (1.033) using intercept from LD score regression^7^.

**^3^** In the combined sample (adults and children), the unaffected parents of affected children and the unaffected children of unaffected parents were removed from the control list.

**^4^** Mor & Barn, Blood donors and Bergen were combined in control sample.

**Supplementary Table 9. Individual neuropsychiatric CNV ADHD association in the Norwegian sample subgroups.**

| **Locus** | **adults and children –**  **aff/ctrl carrier freq (%) ^1^** | **MoBa children -**  **aff/ctrl carrier freq (%)** | **MoBa adults –**  **aff/ctrl carrier freq (%)** | **Bergen adults –**  **aff/ctrl carrier freq (%)** |
| --- | --- | --- | --- | --- |
|  | **OR (95% CI), P corrected ^2^** | **OR (95% CI), P corrected ^2^** | **OR (95% CI), P corrected ^2^** | **OR (95% CI), P corrected ^2^** |
| 1q21.1 - distal - deletion | 0.0928 / 0.0234 | 0.0538 / 0.0485 | 0.106 / 0.0246 | 0.230 / 0 |
|  | 3.97 (0.64, 18.59), 0.075 | 1.11 (0.023, 11.22), 1 | 4.32 (0.094, 35.67), 0.24 | Inf (0.021, Inf), 1 |
| 1q21.1 - distal - duplication | 0.247 / 0.0273 | 0.215 / 0.0485 | 0.319 / 0.0287 | 0.230 / 0 |
|  | 9.09 (2.88, 29.46), 9.9 × 10^-5^ | 4.44 (0.83, 23.88), 0.046 | 11.14 (1.86, 48.87), 0.0058 | Inf (0.021, Inf), 1 |
| 2p16.3 (NRXN1) - deletion | 0.0619 / 0.0273 | 0.108 / 0.0364 | 0 / 0.0246 | 0 / 0 |
|  | 2.27 (0.23, 11.92), 0.27 | 2.96 (0.25, 25.85), 0.24 | 0 (0, 22.04), 1 | 0 (0, Inf), 1 |
| 15q11.2 - deletion | 0.371 / 0.285 | 0.431 / 0.340 | 0.319 / 0.303 | 0.230 / 0 |
|  | 1.31 (0.64, 2.42), 0.39 | 1.27 (0.50, 2.86), 0.53 | 1.05 (0.21, 3.20), 0.77 | Inf (0.021, Inf), 1 |
| 15q13.3 (BP4 & BP4.5 - BP5) - deletion | 0.217 / 0.0273 | 0.323 / 0.0485 | 0.106 / 0.0328 | 0 / 0 |
|  | 7.95 (2.38, 26.58), 4.6 × 10^-4^ | 6.67 (1.58, 32.19), 0.0047 | 3.24 (0.073, 24.22), 0.30 | 0 (0, Inf), 1 |
| 16p11.2 distal - deletion | 0.0309 / 0.0156 | 0.0538 / 0.0364 | 0 / 0.0164 | 0 / 0 |
|  | 1.98 (0.040, 20.06), 0.46 | 1.48 (0.028, 18.43), 0.56 | 0 (0, 39.29), 1 | 0 (0, Inf), 1 |
| 16p11.2 proximal - deletion | 0.0928 / 0.0312 | 0.108 / 0.0121 | 0.106 / 0.0328 | 0 / 0 |
|  | 2.98 (0.51, 12.42), 0.12 | 8.88 (0.46, 522.32), 0.094 | 3.24 (0.073, 24.22), 0.30 | 0 (0, Inf), 1 |
| 16p11.2 proximal - duplication | 0.217 / 0.0390 | 0.269 / 0.0606 | 0.106 / 0.0492 | 0.230 / 0 |
|  | 5.56 (1.80, 16.2), 0.0018 | 4.45 (1.022, 19.33), 0.026 | 2.16 (0.051, 14.63), 0.40 | Inf (0.021, Inf), 1 |
| 16p12.1 - deletion | 0.0928 / 0.0702 | 0.0538 / 0.0849 | 0 / 0.0738 | 0.461 / 0 |
|  | 1.32 (0.25, 4.54), 0.73 | 0.63 (0.014, 4.94), 1 | 0 (0, 5.90), 1 | Inf (0.15, Inf), 0.51 |
| 16p13.11 - duplication | 0.402 / 0.172 | 0.538 / 0.206 | 0.213 / 0.168 | 0.230 / 0.282 |
|  | 2.35 (1.16, 4.45), 0.011 | 2.62 (1.07, 6.07), 0.024 | 1.27 (0.15, 4.88), 0.68 | 0.82 (0.010, 64.3), 1 |
| 17p12 - deletion | 0.0619 / 0.0273 | 0.0538 / 0.0485 | 0.106 / 0.0246 | 0 / 0 |
|  | 2.27 (0.23, 11.92), 0.27 | 1.11 (0.023, 11.22), 1 | 4.32 (0.094, 35.67), 0.24 | 0 (0, Inf), 1 |
| 17q12 - duplication | 0.0928 / 0.0390 | 0.161 / 0.0485 | 0 / 0.0451 | 0 / 0 |
|  | 2.38 (0.42, 9.25), 0.18 | 3.33 (0.49, 19.71), 0.13 | 0 (0, 10.34), 1 | 0 (0, Inf), 1 |
| 22q11.21 - deletion | 0.124 / 0 | 0.215 / 0.0243 | 0 / 0 | 0 / 0 |
|  | Inf (5.24, Inf), 2.0 × 10^-4^ | 8.89 (1.27, 98.36), 0.014 | 0 (0, Inf), 1 | 0 (0, Inf), 1 |
| 22q11.21 - duplication | 0.309 / 0.144 | 0.377 / 0.0606 | 0 / 0.160 | 0.691 / 0 |
|  | 2.15 (0.95, 4.41), 0.040 | 6.23 (1.70, 24.92), 0.0028 | 0 (0, 2.57), 0.41 | Inf (0.34, Inf), 0.26 |
| **Combined neuropsychiatric CNVs** | **2.54 / 0.971** | **3.07 / 1.16** | **1.59 / 1.03** | **2.30 / 0.282** |
|  | **2.66 (2.04, 3.43), 7.8 × 10^-12^** | **2.69 (1.89, 3.78), 5.9 × 10^-8^** | **1.56 (0.86, 2.63), 0.11** | **8.33 (1.18, 362.80), 0.017** |

^1^ In the combined sample (adults and children), the unaffected parents of affected children and the unaffected children of unaffected parents were removed from the control list.

^2^ Odds ratio (OR), 95% confidence interval (95% CI) and P-value are estimated using Fisher exact to test for increased burden of the neuropsychiatric CNVs in the ADHD cases compared with controls in the Norwegian samples. The P values were adjusted with a correction factor (1.033) using intercept from LD score regression^7^.

aff/ctrl carrier freq (%) - affected/control carrier frequency. Refer to Supplementary Table 8 for counts.

**SUPPLEMENTARY FIGURES**

**Supplementary Figure 1. Neuropsychiatric CNVs found in Icelandic and Norwegian ADHD cases or controls. Chromosome, start and stop in human genome build 18 (hg18-NCBI36).**

1q21.1 distal – deletion and duplication


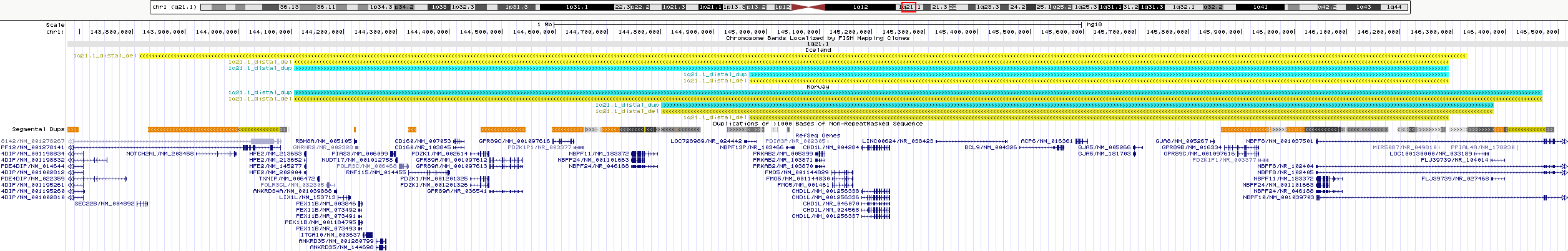


2p13.3 (NRXN1) – deletion


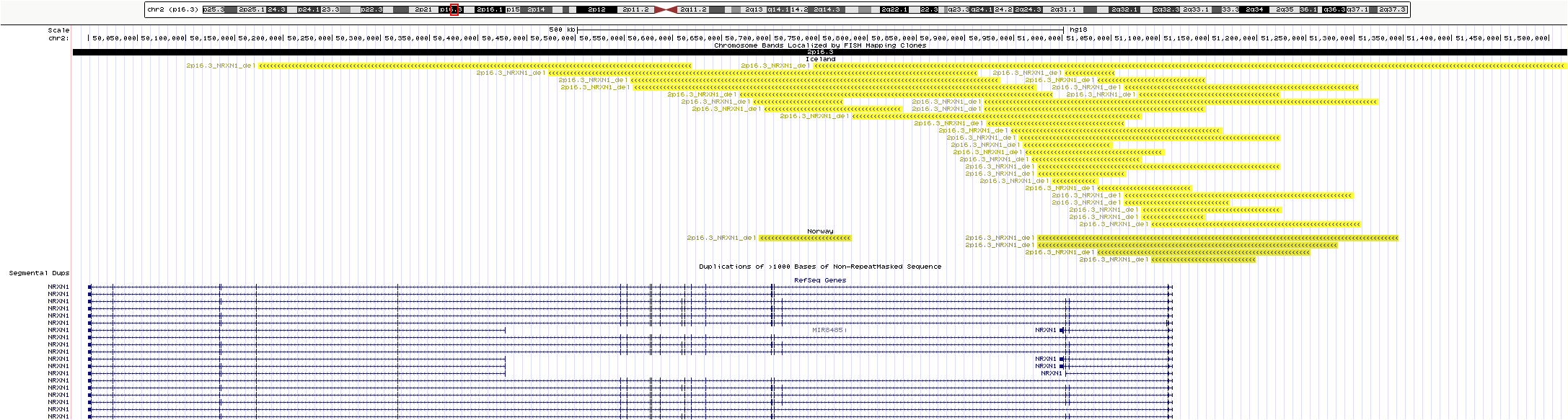


3q29 – deletion


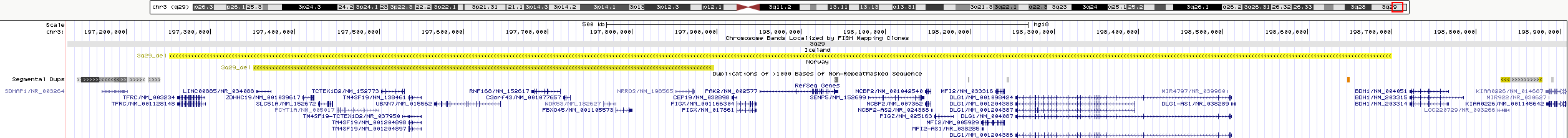


7q11.23 (WBS) – duplication


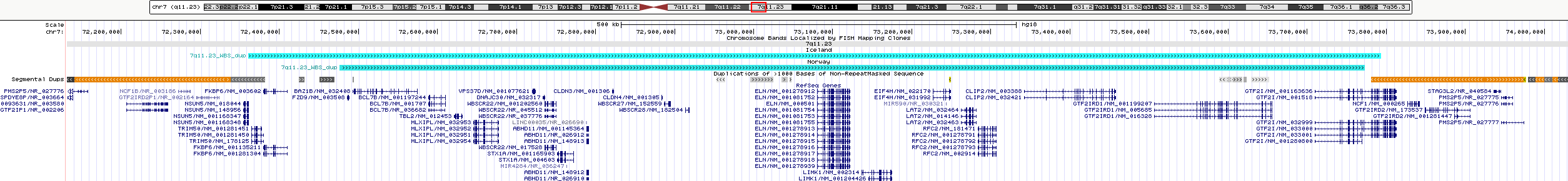


7q36.3 (VIPR2) – duplication


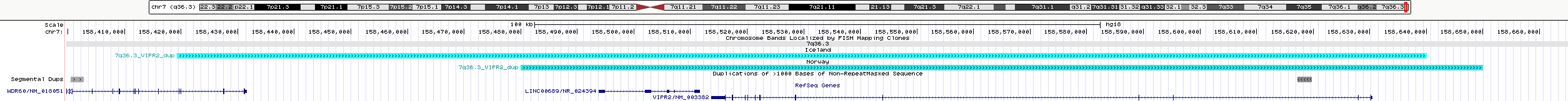


15q11.2 – deletion


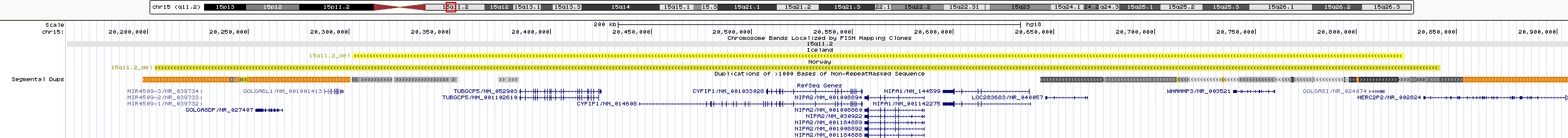


15q11.2-13.1 – duplication


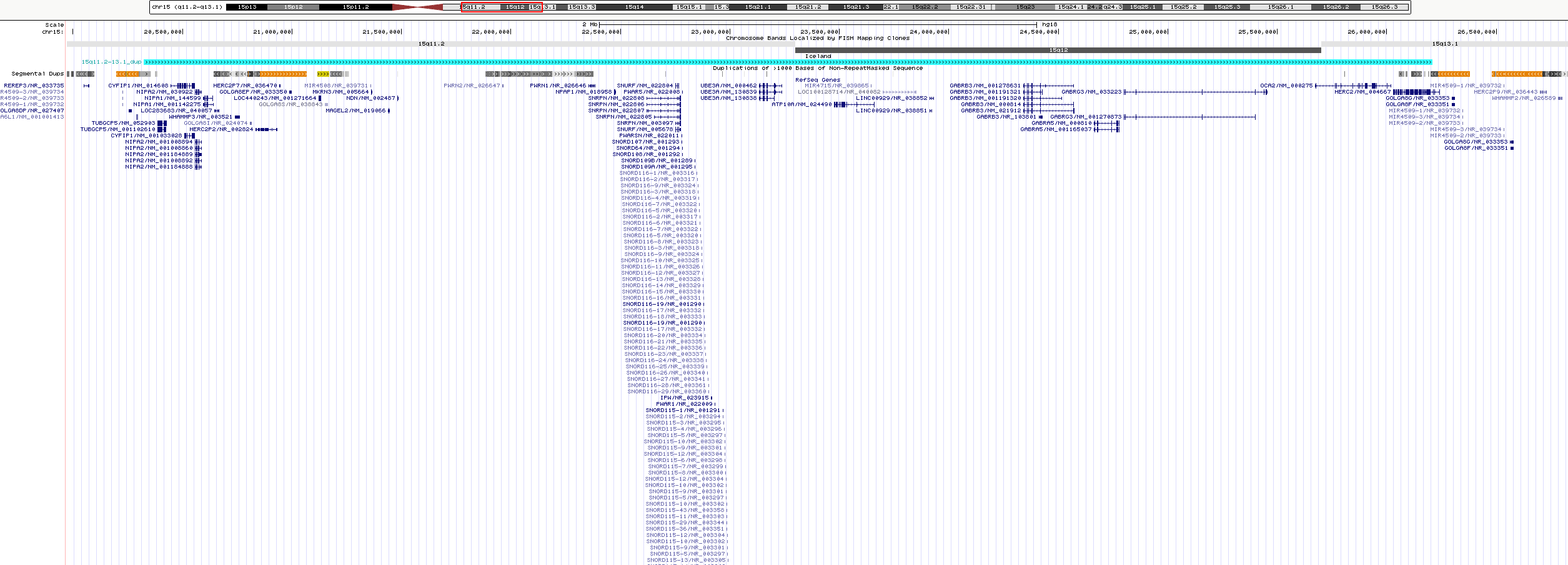


15q13.3 (BP4 & BP4.5 - BP5) – deletion


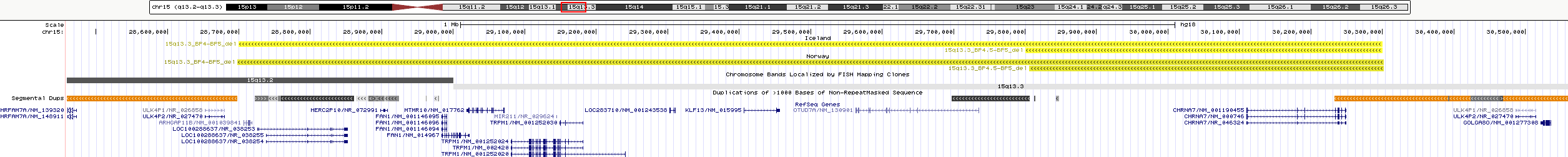


16p11.2 distal – deletion


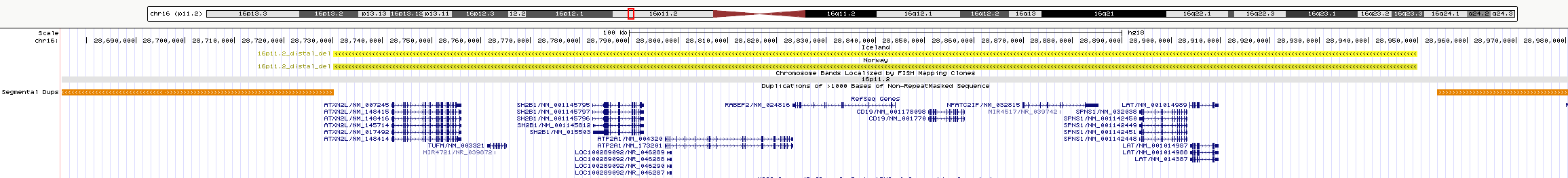


16p11.2 proximal – deletion and duplication


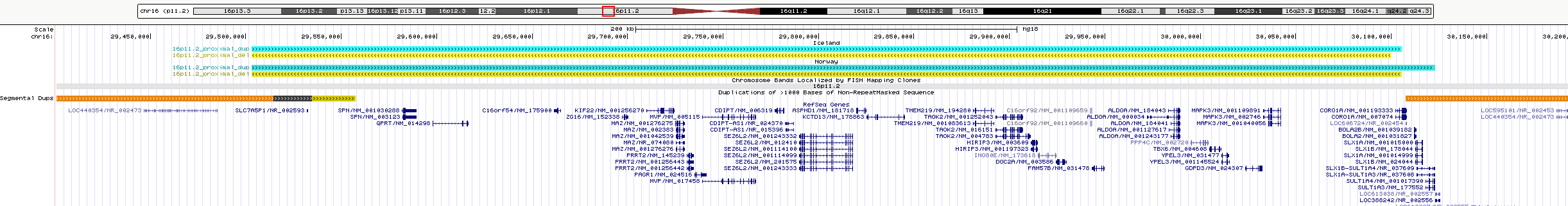


16p12.1 – deletion


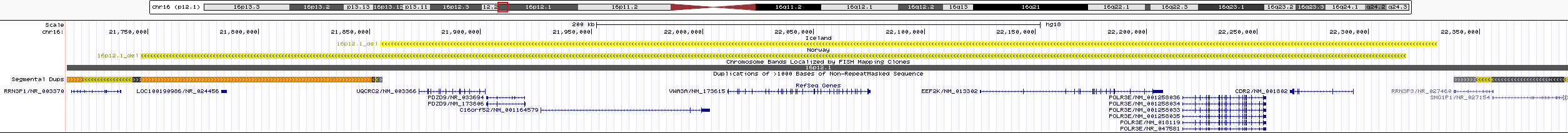


16p13.11 – duplication


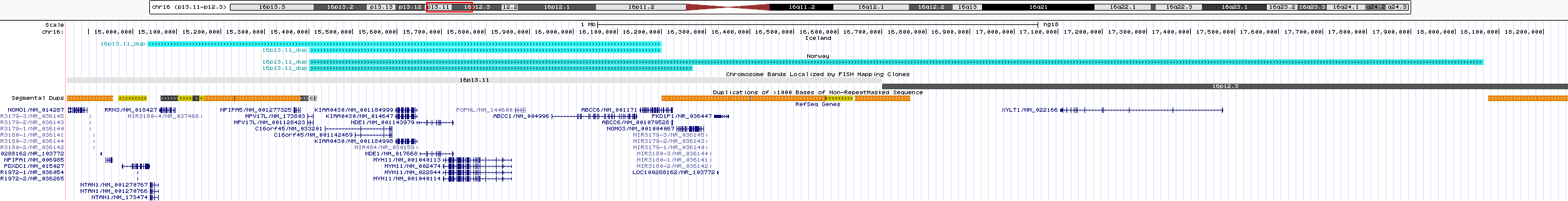


17p12 – deletion


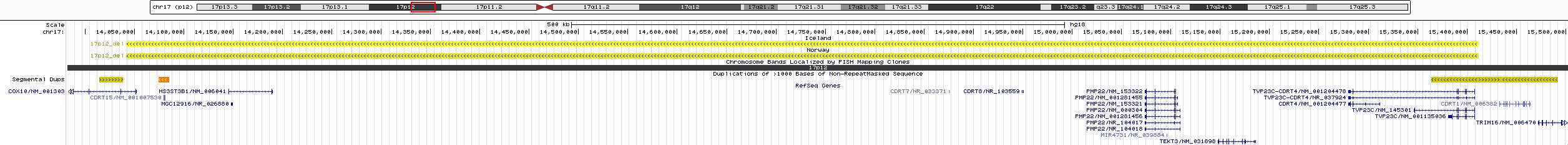


17q12 – deletion and duplication


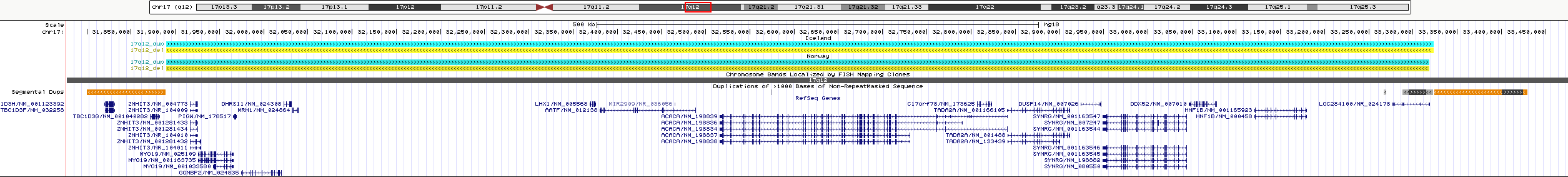


22q11.21 – deletion and duplication


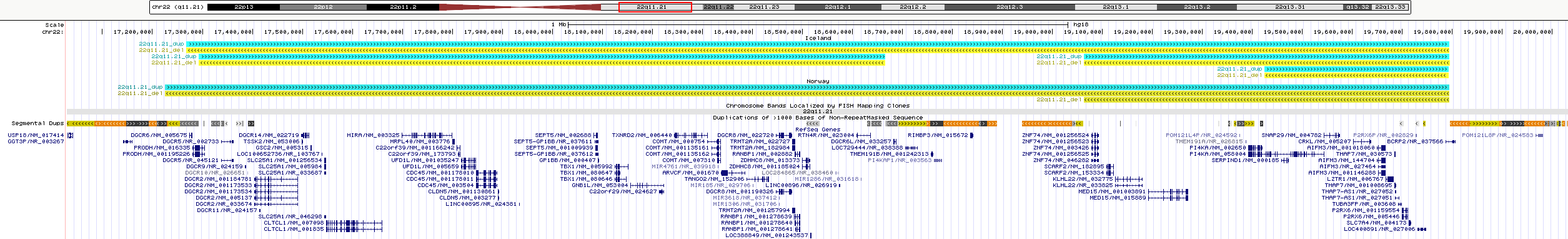


**Supplementary Figure 2. Estimate of minimun CNV population frequency required to detect association.**

The dashed line indicates that we have 80% power to detect an OR of above 3.9 at a CNV population frequency of at least 0.018%.


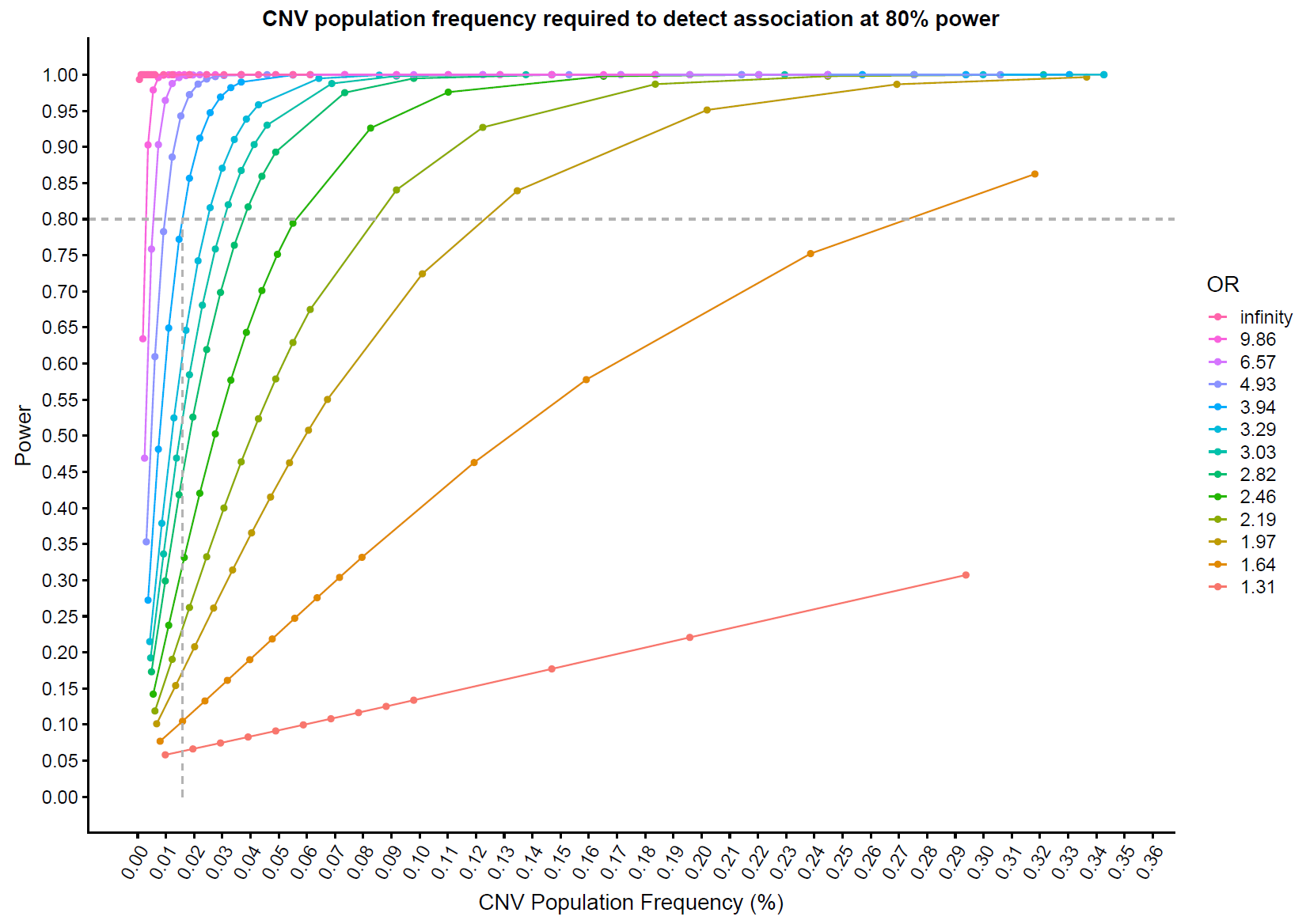


**REFERENCES**

1. Malhotra D, Sebat J. CNVs: harbingers of a rare variant revolution in psychiatric genetics. *Cell* 2012; **148**(6)**:** 1223-1241.

2. Rees E, O'Donovan MC, Owen MJ. Genetics of schizophrenia. *Current Opinion in Behavioral Sciences* 2015; **2:** 8-14.

3. Pinto D *et al.* Convergence of genes and cellular pathways dysregulated in autism spectrum disorders. *Am J Hum Genet* 2014; **94**(5)**:** 677-694.

4. Marshall CR *et al.* Contribution of copy number variants to schizophrenia from a genome-wide study of 41,321 subjects. *Nat Genet* 2017; **49**(1)**:** 27-35.

5. Rees E *et al.* Evidence that duplications of 22q11.2 protect against schizophrenia. *Mol Psychiatry* 2014; **19**(1)**:** 37-40.

6. Wenger TL *et al.* 22q11.2 duplication syndrome: elevated rate of autism spectrum disorder and need for medical screening. *Mol Autism* 2016; **7:** 27.

7. Bulik-Sullivan BK *et al.* LD Score regression distinguishes confounding from polygenicity in genome-wide association studies. *Nat Genet* 2015; **47**(3)**:** 291-295.
